# Dynamic reworking of marine diatom endometabolomes in response to temperature and a model bacterium

**DOI:** 10.1128/msystems.01036-25

**Published:** 2025-12-15

**Authors:** Malin Olofsson, Mario Uchimiya, Frank X. Ferrer-González, Jeremy E. Schreier, McKenzie A. Powers, Christa B. Smith, Arthur S. Edison, Mary Ann Moran

**Affiliations:** 1 Department of Marine Sciences, University of Georgia 730301, Athens, Georgia, USA; 2 Department of Aquatic Sciences and Assessment, Swedish University of Agricultural Sciences 166469, Uppsala, Sweden; 3 Complex Carbohydrate Research Center, University of Georgia 123423, Athens, Georgia, USA; 4 Department of Biochemistry and Molecular Biology, University of Georgia 174518, Athens, Georgia, USA; Stellenbosch University, Stellenbosch, South Africa

**Keywords:** diatoms, endometabolites, temperature acclimation, bacteria, co-culture

## Abstract

A large annual carbon flux occurs through the surface ocean’s labile dissolved organic carbon (DOC) pool, with influx dominated by phytoplankton-derived metabolites and outflux by heterotrophic bacterioplankton uptake. We addressed the dynamics of this carbon flow between microbial primary and secondary producers through analysis of the *Thalassiosira pseudonana* CCMP1335 endometabolome, a proxy for the labile DOC released upon phytoplankton lysis, as temperature and bacterial presence were altered. Diatom strains acclimated at one of three different temperatures (14°C, 20°C, or 28°C) were cultured either axenically or with the bacterium *Ruegeria pomeroyi* DSS-3, and their endometabolites analyzed by NMR. Median concentration variation between conditions was ~1.5-fold across all identified endometabolites. Those with roles as osmolytes varied most, exhibiting concentration differences up to 170-fold across conditions with the largest variations triggered by the presence/absence of the heterotrophic bacterium. Differential expression observed for diatom metabolite synthesis pathways suggested changes in synthesis rates as a mechanism for endometabolome remodeling. Consistent with expectations of high turnover by heterotrophic bacteria, endometabolite mean lifetimes in a DOC pool were <2 h to 12 h.

**IMPORTANCE:**

The role of labile DOC in the transfer of marine carbon between phytoplankton and heterotrophic bacteria was first recognized 40 years ago, yet the identity and dynamics of phytoplankton metabolites entering the labile DOC pool are still poorly known. Using metabolome and transcriptome profiling, we found highly variable composition and concentration of diatom endometabolites, depending on growth conditions and arising over time frames as short as a single growth cycle. This strong response to external conditions, both biotic and abiotic, suggests that the chemical composition of phytoplankton intracellular pools released during lysis shift with ocean conditions. As phytoplankton cell lysis is one of the largest sources of labile dissolved compounds in the ocean, dynamic compositional changes in the metabolites released to heterotrophic bacteria have implications for the fate of surface ocean carbon.

## INTRODUCTION

In the microbe-metabolite network of the surface ocean, a large fraction of heterotrophic bacterial production is supported by the organic carbon released by marine phytoplankton into the dissolved organic carbon (DOC) pool (1, 2). The mechanisms by which labile metabolites enter this carbon reservoir fall into three categories: release from living phytoplankton (both diffusive and active processes); release from heterotrophs (secondary producer exudates and waste) (3); and release from phytoplankton cell rupture (zooplankton grazing, viral lysis, and senescence) (4–6). The last of these mechanisms liberates a mixed suite of internal metabolites from phytoplankton cells that can include polysaccharides (7), organic sulfur compounds (8), sugars, amino acids (2), and osmolytes (9). Quantitatively, phytoplankton endometabolite release by cell rupture mechanisms is estimated to account for ~40% of labile DOC inputs to surface seawater (1).

Once metabolites are released into the DOC pool, their uptake and processing by bacteria represents a major carbon flux at the global scale. In model organism studies (10, 11) and natural bacterial communities (12), marine bacteria metabolize components of the mixed metabolite pool selectivity, sometimes referred to as “substrate preference,” likely driven by genomic constraints, enzyme kinetic properties, or regulatory processes. At a broad taxonomic level, members of the *Roseobacteraceae* take up a range of organic acids and sulfur-containing metabolites (13, 14), whereas *Flavobacteriaceae* dominate polysaccharide degradation (10, 13, 15), and *Gammaproteobacteria* target peptides, polysaccharides, and monosaccharides (12). Some studies suggest substrate energy content as a driver of preferential bacterial uptake (16), while others propose a metabolic specialization predictable from genome content (11, 17). Regardless of the underlying mechanism, the chemical composition of intracellular pools released by phytoplankton is likely to impact downstream processing by heterotrophic bacterioplankton communities.

As ocean temperatures shift on annual and decadal scales (18), the consequences to phytoplankton endometabolome composition are hard to anticipate. Here, we ask whether acclimation of diatom strains at different temperatures affects the accumulation of their internal metabolites, and whether there are effects from the presence of heterotrophic bacteria that would be missed in axenic studies. From previous research, temperature shifts have been shown to alter phytoplankton physiology by changes in cell size (19, 20), shifts in chlorophyll *a* concentration (21, 22), and alterations of protein content (21). Furthermore, increased temperature may impact the release of endometabolites by cell breakage, for example, by altering timing of viral infections in phytoplankton blooms (23) or rates of zooplankton feeding (24). Environmentally driven changes in endometabolite concentration or composition would likely have repercussions for bacterial processing of the labile DOC pool.

In this study, the marine diatom *Thalassiosira pseudonana* CCMP1335 was acclimated over 3 months at three temperatures, one at the diatom’s optimal growth temperature (20°C) (22), one below (14°C), and one above (28°C). Following acclimation, diatom co-cultures were inoculated with the marine bacterium *Ruegeria pomeroyi* DSS-3 or were left axenic. Diatom endometabolites were examined by three methods: identification and quantification by nuclear magnetic resonance (NMR) spectroscopy, expression analysis of metabolite biosynthesis pathways, and lability assessment via bacterial drawdown assays. The convergence of these data sets provides evidence of considerable temperature-related restructuring of diatom endometabolomes, points to an unexpectedly strong impact of heterotrophic bacteria on endometabolome composition, and singles out osmolytes as the organic compound class most responsive to conditions in the diatom’s environment.

## RESULTS AND DISCUSSION

### Axenic *T. pseudonana* endometabolomes are restructured during temperature acclimation

**Fig 1:**
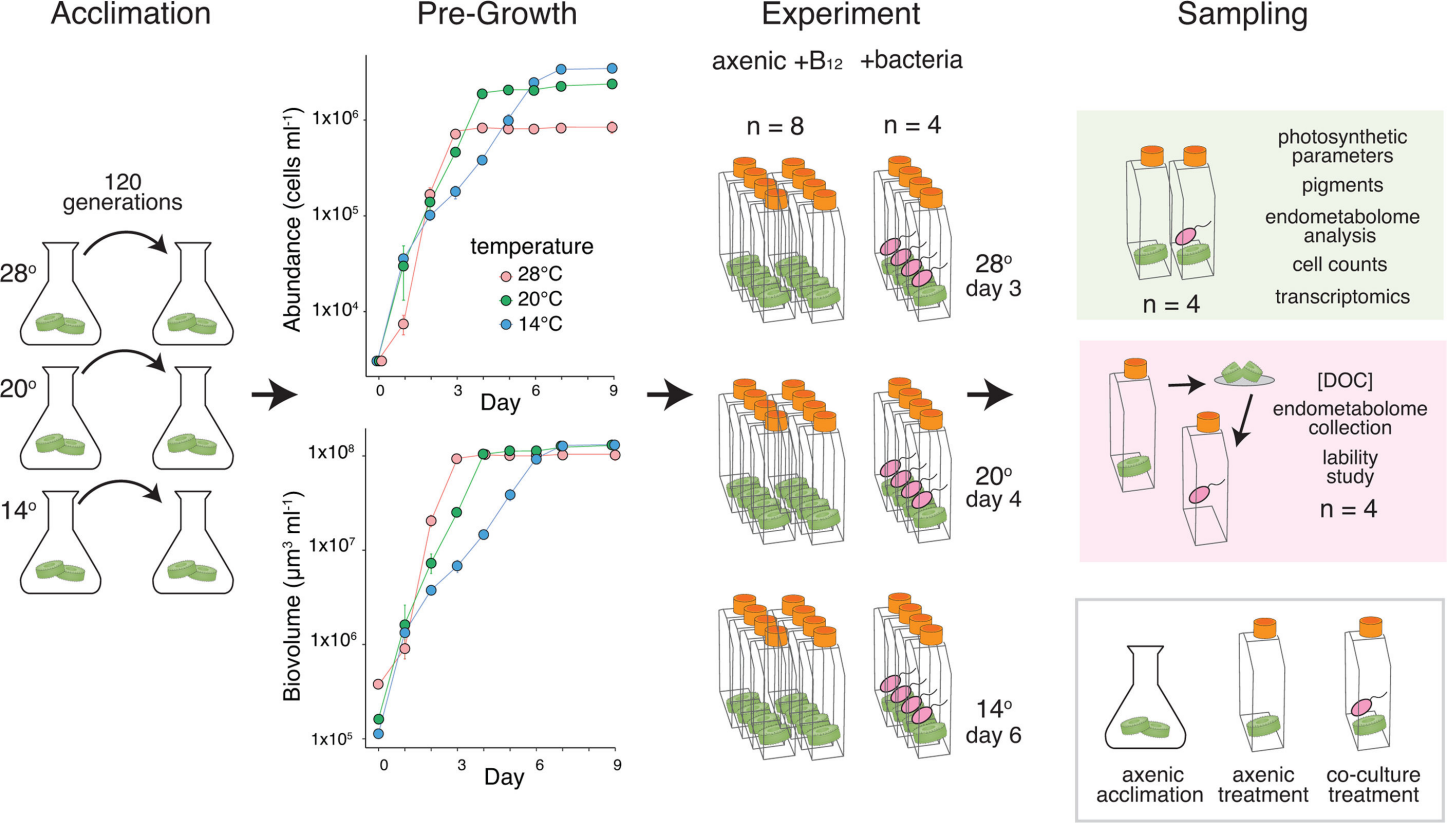
(left to right) Acclimation: *T. pseudonana* cells were temperature acclimated for 120 generations. Pre-Growth: Cell abundance (top) and biovolume (bottom) were measured in axenic cultures following acclimation to determine the timing of late exponential phase for each strain (*n* = 2). Error bars indicate ±1 standard deviation; most data points fall within the symbols. Experiment: Temperature acclimated *T. pseudonana* cultures were inoculated with *R. pomeroyi* or remained axenic and were grown to late exponential phase. Sampling: At harvest, diatom cells were collected for physiological measurements, endometabolome analysis, and transcriptomics (green shading). In a separate metabolite lability study (pink shading), axenic diatom endometabolomes were inoculated with *R. pomeroyi* to measure uptake rates. DOC, dissolved organic carbon.

Axenic cultures of the diatom *T. pseudonana* CCMP1335, originally isolated from 20°C coastal water off Long Island, NY, USA (25), were pre-acclimated at 14°C, 20°C, and 28°C (Fig. 1) under replete nutrient and vitamin conditions over 3 months with weekly transfers (~120 generations). These three temperatures represented below optimal, at optimal, and above optimal conditions based on the diatom’s growth rate. Following acclimation, an experiment was established to compare endometabolome composition across the temperatures and in the presence or absence of a heterotrophic bacterium (Fig. 1). Diatom cultures were incubated over one growth cycle at their temperature of acclimation, with or without a co-cultured heterotrophic bacterium, until late exponential phase, then harvested and analyzed for endometabolome composition.

Because exponential growth was offset in time across the temperatures (Fig. 1), consistent with expectations in nutrient replete conditions (26), cultures were harvested at 3 days (28°C), 4 days (20°C), or 6 days (14°C). Cell numbers were higher at 14°C but cell volumes were larger at 28°C (Table S1), the latter in accordance with data on temperature influence on *T. pseudonana* cell size (22, 27) and consistent with reports of diatoms being an exception to the negative size-temperature relationship (20, 28, 29), potentially enhancing storage capabilities in nitrogen-variable environments (30, 31). Because of the cell size differences, diatom biovolume was similar at stationary phase (Fig. 1). Photochemical efficiency measurements were indicative of healthy cells in all treatments (Fv/Fm values > 0.70; Table S1).

**Fig 2:**
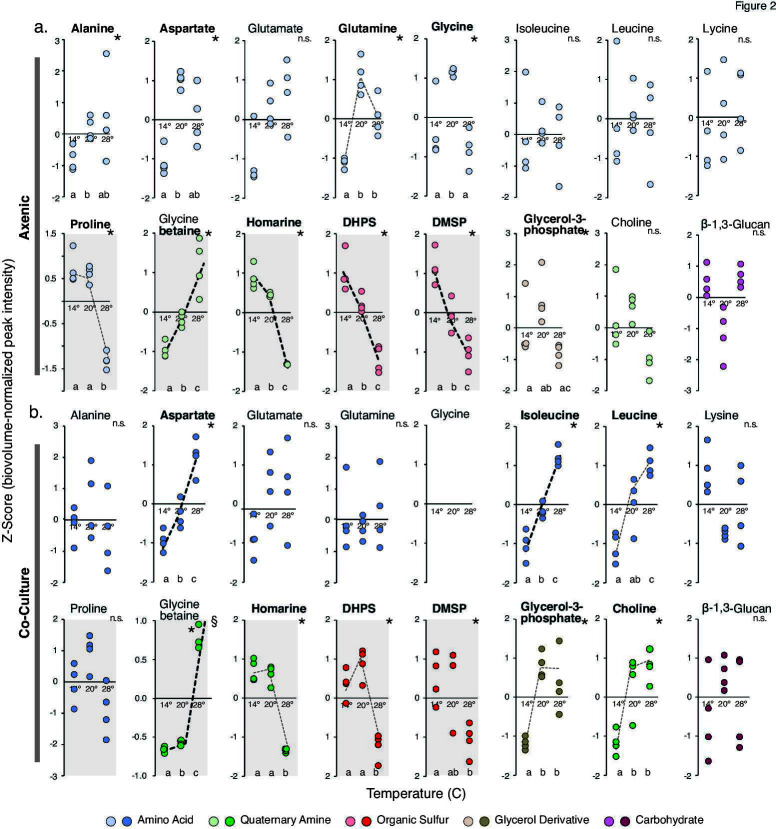
Abundance of endometabolites in temperature acclimated *T. pseudonana* strains. Peak intensity is normalized to diatom biovolume at time of harvest and presented as *Z*-scores for (**a**) axenic cultures and (**b**) co-cultures. Bold compound names indicate significant differences in metabolite concentrations (Kruskal Wallis Test, *P* ≤ 0.05), and unalike lower-case letters at the bottom of each plot indicate which pairwise comparisons are significantly different in the post-hoc comparisons (Mann-Whitney *U* Tests, *P* ≤ 0.05). Dotted lines highlight metabolites that trend with temperature as either a linear (bold dashed lines) or threshold (light dashed lines) response. Osmolytes are indicated by shaded plots. §, one replicate for glycine betaine at 28°C plots above the *Y* axis at 2.4 and is not shown.

Endometabolomes were liberated from the axenic diatom cultures at harvest to characterize intracellular metabolites, serving as proxies for compounds released by excretion or mortality processes in the ocean. Sixteen metabolites were annotated by NMR (Fig. 2a; Table S2). Ten had significantly different concentrations for at least one of the three pairwise temperature comparisons (14°C vs 20°C, 14°C vs 28°C, and 20°C vs 28°C) (Kruskal Wallis with post-hoc pairwise Mann-Whitney *U* tests, *P* ≤ 0.05) (Fig. 2a). We recognized two patterns of temperature responsiveness. In linear responses, concentration changes were consistent along the temperature range (i.e., increases or decreases that correlated with temperature based on the post hoc Mann-Whitney *U* tests). In threshold responses, concentrations exhibited a step change at one point in the temperature range (i.e., one significant increase or decrease across the temperature scale). Considering both linear and threshold patterns, six metabolites showed temperature responsiveness under axenic conditions (Fig. 2a). Glycine betaine and glutamine concentrations were positively related to temperature, and proline, homarine, 2,3-dihydroxypropane-1-sulfonate (DHPS), and dimethylsulfoniopropionate (DMSP) concentrations were negatively related. All but glycine function as osmolytes (9) and typically are found at high concentrations in marine phytoplankton cytosols. For example, previous measures of these compounds in axenic *T. pseudonana* cells ranged from 16 mM to 118 mM (8, 32, 33). Such high concentrations may reflect the metabolites’ cellular roles in addition to regulating osmosis (34), for example, alleviating temperature stress by inhibiting protein denaturation (35). Indeed, osmolyte concentration changes in phytoplankton endometabolomes in response to temperature have been reported previously in both laboratory and field studies (33, 36, 37). Nine amino acids were also present in the axenic *T. pseudonana* endometabolomes (Fig. 2a), one of which showed a positive threshold response (glutamine) and one a negative response (proline).

Overall, compounds dominating the diatom endometabolomes were consistent with those observed in natural phytoplankton communities (8, 32, 33, 38). For example, in a North Pacific Ocean study, glycine betaine and proline were among the most abundant osmolytes; and alanine, aspartate, glutamate, glutamine, and leucine were among the most abundant amino acids (36).

### *T. pseudonana* endometabolomes differ between co-culture and axenic conditions

The *T. pseudonana* endometabolome composition was also analyzed after acclimated strains were grown in co-culture with a heterotrophic marine bacterium (Fig. 2b), as would occur in a natural seawater environment. Diatom cultures to be inoculated with *Ruegeria pomeroyi* DSS-3, a representative of the *Roseobacteraceae* family whose members are frequently found in association with phytoplankton blooms (12), were first stepwise limited by B_12_. This essential vitamin is not synthesized by *T. pseudonana* and must be supplied exogenously by associated marine bacteria in nature. As *R. pomeroyi* was the only source of B_12_ for the diatom, and the diatom was the only source of substrate for the bacterium, a mutual reliance was established in the co-cultures. Similar to axenic cultures, co-cultures were incubated at the diatom’s temperature of acclimation for one growth cycle until harvest. The co-cultured diatoms’ growth rates were slower than in axenic cultures by 12%–21% (Table S1), suspected to be related to the bacterial B_12_ supply rate. This was consistent with the enrichment of the diatom’s B_12_ acquisition protein CBA1 (39) in co-culture transcriptomes compared to their axenic counterparts (Tables S3 to S5).

Only 15 compounds were identified in the co-cultured diatom endometabolomes; glycine was not detected. Three had temperature responses that followed the patterns of the axenic cultures; these were osmolytes glycine betaine (increasing concentration with increasing temperature), and homarine and DHPS (decreasing concentration with increasing temperature). Five others had significant temperature responses that were not observed under axenic conditions; these were three amino acids (aspartate, isoleucine, and leucine), glycerol-3-phosphate, and choline, which all increased in concentration with increasing temperature (Fig. 2b).

**Fig 3:**
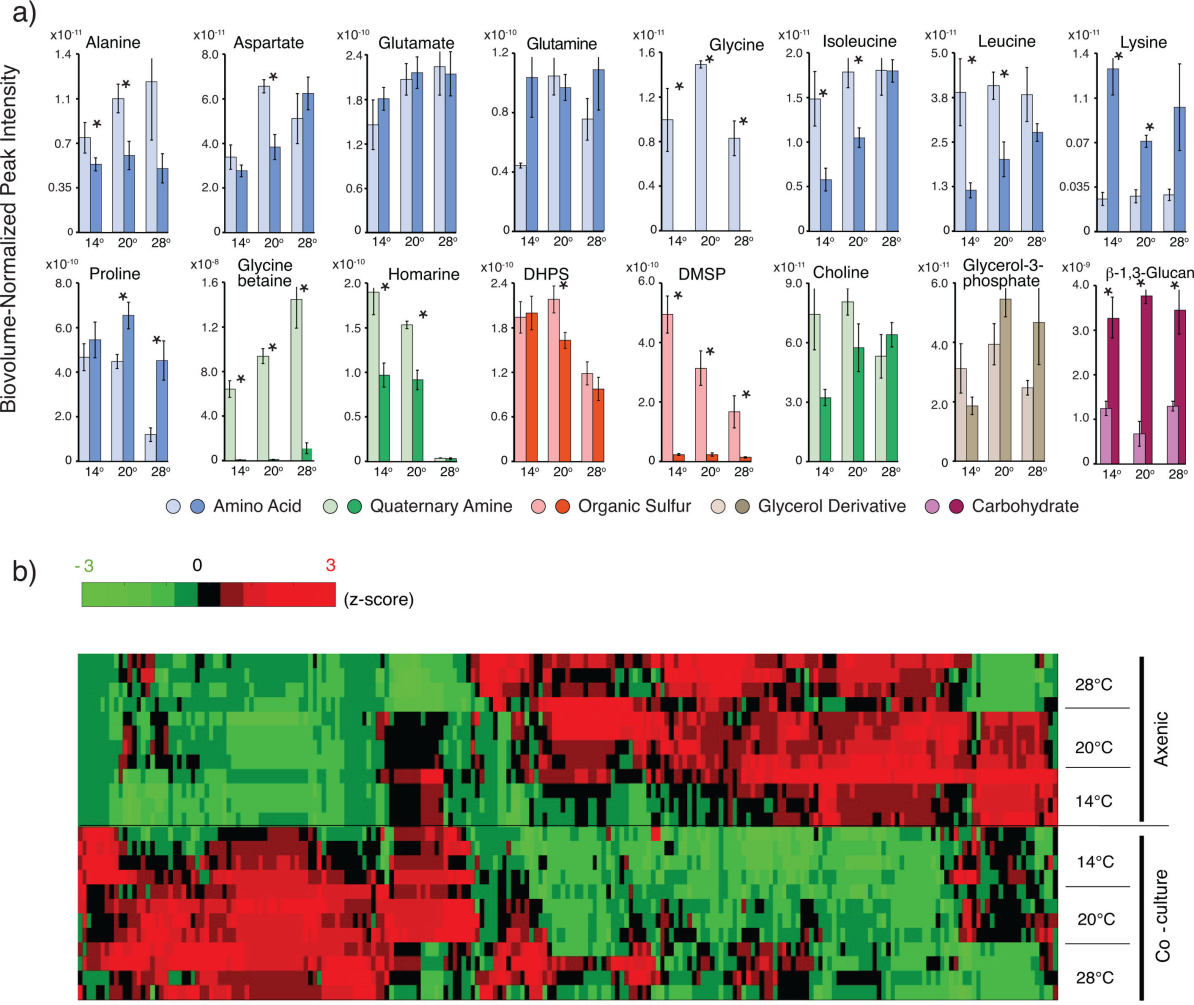
(**a**) Comparison of peak intensity for annotated endometabolites in temperature-acclimated *T. pseudonana* strains from axenic cultures (light bars) and co-cultures (dark bars). Error bars show ±1 standard deviation, *n* = 4. Asterisks above a co-culture/axenic pair denote differences in concentration based on Student’s *t*-tests with Benjamini-Hochberg multiple comparison corrections (*n* = 4, *P* ≤ 0.05). (**b**) Comparison of endometabolite peak intensity for the full suite of peaks in the NMR spectra (i.e., whether or not they were assigned to a known compound) from axenic cultures (top) and co-cultures (bottom). Values shown are *z*-scores of peak intensity following normalization to diatom biovolume at time of harvest.

The concentrations of endometabolome compounds differed substantially between the axenic and co-cultured diatoms, with largest changes again seen for osmolytes. Twelve of the 16 identified metabolites had statistically different concentrations for at least one acclimation temperature (Wilcoxon Rank Sum Test, Bonferroni multiple tests correction, *P* ≤ 0.05; Fig. 3a). Three had higher concentrations in the co-cultures compared to axenic: β-1,3-glucan (fourfold, average of three temperatures), lysine (fourfold), and proline (twofold). Nine had lower concentrations in co-cultures, with the most extreme differences found for glycine betaine (97-fold), DMSP (16-fold), and glycine (not detected in co-culture endometabolomes) (Fig. 3a). A further comparison of the full suite of peaks in the NMR spectra (i.e., whether or not they were annotated as a known compound) similarly showed that interfacing with the bacterium for one growth cycle altered concentrations of the majority of endometabolites (Fig. 3b). These data portray diatom endometabolomes as highly dynamic in composition and decidedly responsive to environmental conditions linked to both temperature changes and interactions with bacteria. We explored a possible temperature acclimation mechanism with transcriptome data from the *T. pseudonana* strains.

### *T. pseudonana* metabolite biosynthesis pathways have temperature-specific expression patterns

**Fig 4:**
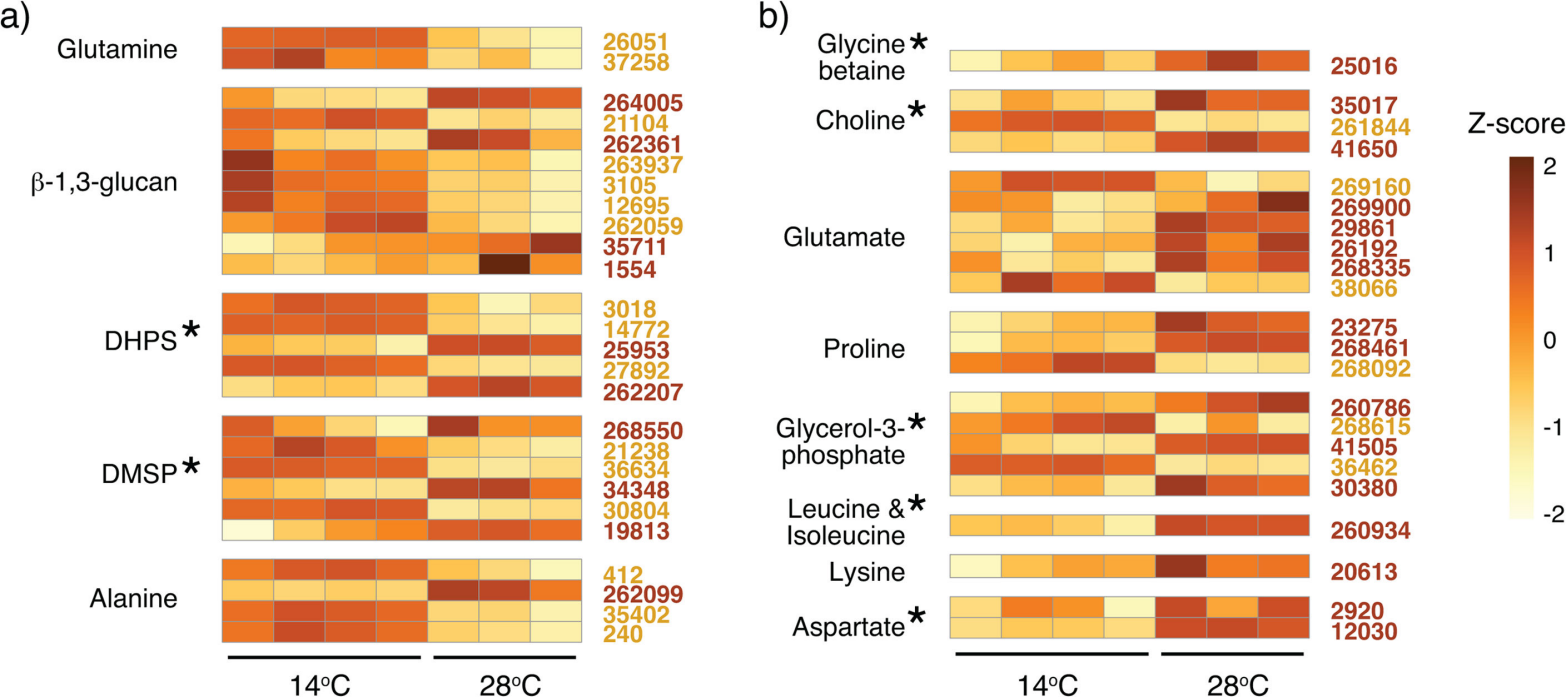
Co-cultured *T. pseudonana* gene expression of endometabolite biosynthetic pathways with reactions biased toward enriched expression in 14°C transcriptomes or 28°C transcriptomes, normalized as *Z*-scores. Each row represents a gene in a tentative biosynthetic pathway, and each column represents a treatment replicate. Gene locus tags in gold font are enriched at 14°C and brown are enriched at 28°C. (**a**) Pathways with the majority of significant genes enriched at 14°C. (**b**) Pathways with the majority of significant genes enriched at 28°C. Asterisks indicate significant differences in endometabolite concentration between 14°C and 28°C as shown in Fig. 2 that are consistent with pathway expression. One replicate of the 28°C-acclimated cultures was an outlier and is not included.

Changes in the diatom endometabolome pools in response to variations in environmental conditions, regardless of ultimate mechanism, must result from a proximal effect on input rates (i.e., metabolite biosynthesis) and/or on output rates (e.g., metabolites repurposed internally or released externally). To address the former, transcriptomes of co-cultured *T. pseudonana* were compared among the temperature-acclimated strains. Of the 11,675 predicted genes, ~7,000 had significantly altered relative expression between the 14°C and 28°C transcriptomes (Table S6). For 13 metabolite biosynthesis pathways tentatively identified in the genome (Table S7), 5 had more genes enriched at 14°C (up to 30-fold; DESeq2, *P*adj ≤ 0.05) and 8 had more genes enriched at 28°C (up to 2-fold; DESeq2, *P*adj ≤ 0.05) (Fig. 4). The pairwise comparisons with the 20°C transcriptome were largely intermediate to these results for these temperature end points (Fig. S1).

We observed some consistency between the direction of expression changes in tentative metabolite synthesis pathways (Fig. 4) and the direction of concentration shifts in the diatom’s endometabolome (Fig. 2b). DHPS and DMSP had greater biosynthetic pathway enrichment and higher endometabolome concentrations at 14°C relative to 28°C. Aspartate, leucine, isoleucine, glycine betaine, glycerol-3-phosphate, and choline had higher biosynthetic pathway enrichment and higher concentrations at 28°C relative to 14°C. These matches between metabolite concentrations and directional change in gene expression suggest regulation of synthesis pathways as one mechanism of temperature acclimation. Other hints of drivers of metabolite alterations based on transcriptome data include altered metabolite exudation from photosynthetic overflow or photorespiration (6), consistent with significant enrichment of transcripts for two central photorespiration genes: phosphoglycolate phosphatase (1.4-fold) and (S)-2-hydroxy-acid oxidase (1.4-fold) in the 28°C acclimated strain; altered levels of reactive oxygen stress, evidenced as enrichment in catalase and superoxide dismutase transcripts (14°C strain; 1.3- and 1.8-fold); and changes in nutrient requirements, evidenced as enrichment of four of the five ammonium transporters (28°C transcriptome; 1.3- to 1.9-fold) (Table S6).

### Bacterial drawdown rates indicate lability of *T. pseudonana* endometabolites

**Fig 5:**
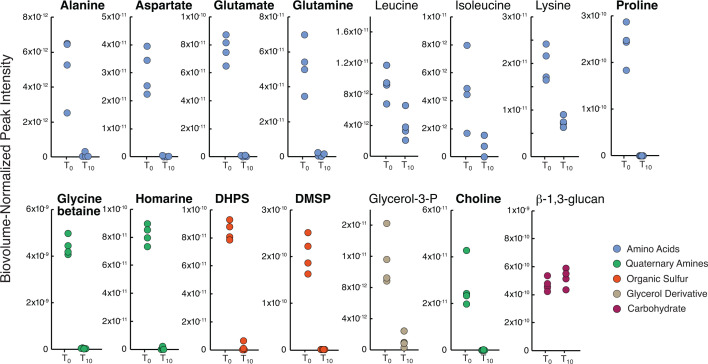
Endometabolite lability experiment measuring *R. pomeroyi* assimilation of compounds in lysates from temperature-acclimated axenic diatoms. Metabolite concentrations were measured at the time of inoculation (*T*_0_) and after 10 h (*T*_10_). Those with bold names were drawn down to <2% of initial concentrations. Data are from the 20°C-acclimated diatom treatment; drawdown results at the other two temperatures are consistent (Fig. S2). Endometabolite peak intensity is normalized to diatom biovolume at the time of harvest. Concentrations at inoculation compared to 10 h are significantly different for all metabolites except β-1,3-glucan (student’s *t*-test with Benjamini-Hochberg correction, *P* < 0.05; *n* = 4).

*R. pomeroyi* abundance increased an average of 5.6-fold (±0.8) in *T. pseudonana* co-cultures between inoculation and late exponential phase harvest. As phytoplankton endometabolites were the only source of substrates available to the bacterium, we conducted drawdown experiments to determine which of the identified metabolites were supporting *R. pomeroyi* growth. Cell lysates collected from a second set of axenic flasks (*n* = 4 for each acclimation temperature; Fig. 1) were diluted to 204 ± 12 µM C, brought to 30°C, and inoculated with *R. pomeroyi*. Concentrations quantified by NMR analysis at the time of inoculation and again after 10 h indicated decreases in all endometabolites except β-1,3-glucan, a component of the storage molecule laminarin (Fig. 5). This metabolite can be readily degraded by other marine bacteria (12, 40), but the *R. pomeroyi* genome lacks a laminarinase gene.

We calculated a mean lifetime for the metabolites, i.e., the average length of time a spent medium metabolite stayed in the DOC pool before uptake (calculated as 1/*l*, where *l* is the specific drawdown rate). For the 10 metabolites with final concentrations of ≤2% of initial after 10 h (Fig. 5), mean lifetimes were 2 h or shorter, depending on the time at which complete drawdown occurred. Thus, alanine, aspartate, glutamate, glutamine, proline, glycine betaine, homarine, DHPS, DMSP, and choline were quickly assimilated from the substrate pool. The rapid metabolism of these diatom-derived compounds by *R. pomeroyi* is consistent with earlier findings that they represent major currencies of surface ocean carbon flux (10, 41–43). Metabolites isoleucine, glycerol-3-phosphate, lysine, and leucine had mean lifetimes of 4, 5, 10, and 12 h, respectively, reflecting drawdown to concentrations between 40% and 10% of initial at the 10 h sample (Fig. S2). Metabolites in this second group are less likely to support *R. pomeroyi* growth when competing with other bacterial species for labile DOC.

Bacteria in the lability assay assimilated metabolites within the same timeframe regardless of diatom acclimation temperature. Thus, as expected, lability was not affected by acclimation temperature of the strain providing the cell lysate (22) (student’s *t*-test, *P* ≤ 0.05; *n* = 3 or 4). There was one exception, however, in the 14°C endometabolomes in which DHPS was drawn down to only 30% of initial concentration at 14°C compared to ≤2% of initial concentrations for 20°C and 28°C lysates (Fig. S2). No other metabolites had lower draw down of the 14°C cell lysates, ruling out an inhibitory compound in the endometabolome. However, the higher concentration of DHPS in the 14°C endometabolomes compared to other temperatures (Fig. 2a) would increase the residence time of DHPS without a change in the specific bacterial uptake rate. Alternatively, other metabolites whose concentrations also peaked at 14°C may have been prioritized, lowering *R. pomeroyi*’s uptake rate of DHPS; candidate molecules include proline, homarine, and DMSP.

### A convergence of evidence points to dynamic compositional shifts in the *T. pseudonana* endometabolome

Acclimation of axenic *T. pseudonana* at three different temperatures over ~120 generations resulted in median endometabolite concentration differences of 1.5-fold across all pairwise comparisons (i.e., 14°C vs 20°C, 14°C vs 28°C, and 20°C vs 28°C) for the 16 metabolites; 46% were higher at the higher temperature, 54% were lower (*n* = 48). The presence of a bacterium in the *T. pseudonana* culture had an even larger median difference of twofold; 37% were lower than axenic, 63% were higher. In these comparisons, osmolytes were the most responsive (Fig. 2 and 3), not surprising given the many roles played by osmolytes in maintaining salt homeostasis (9), preventing protein denaturation (35), scavenging reactive oxygen species (44), maintaining membrane fluidity and cryoprotection (35), and encouraging growth of beneficial bacteria (45, 46). The specific metabolites with the largest differences across all comparisons were glycine betaine and DMSP (between co-culture and axenic conditions, 167-fold and 16-fold, respectively) and homarine (between acclimation temperatures, 33-fold). Similar concentration dynamics have been documented previously, for example, for glycine betaine in natural phytoplankton endometabolomes (36) and DMSP in sea-ice diatom endometabolomes (33).

Glycine also exhibited an extreme concentration shift in *T. pseudonana* endometabolomes, being present in axenic culture but undetectable in co-cultures (Fig. 3a). The strong response of this particular metabolite in the presence of *R. pomeroyi* is interesting in light of evidence that exogenous glycine can have diverse effects on bacterial physiology, including enhancement of oxidative stress (47) and inducement of morphological changes in membrane lipopolysaccharides (48). Taken together, dynamic changes in diatom endometabolite composition driven by temperature shifts and bacterial influence are consistent with previous observations of dynamic endometabolite changes related to vitamin limitation (49), growth stage (15), and salinity (33).

### Conclusions

Phytoplankton endometabolites enter the labile DOC pool through two main mechanisms: through selective release from healthy cells (6), and through cell membrane disruption in dying cells by zooplankton ‘sloppy feeding’ and viral lysis (3, 5, 50). The second mechanism, which releases a largely intact pool of mixed endometabolites into seawater, has been estimated to account for half of the labile phytoplankton-derived compounds released directly into the surface ocean DOC pool (51). Among the 16 diatom endometabolites identified here, two have been proposed as major substrates for marine bacterial growth: glycine betaine, estimated to account for up to 35% of heterotrophic bacterial carbon demand (52), and DMSP estimated to account for up to 10% (53, 54). These two compounds are also osmolytes and, along with other endometabolites in this chemical classification, exhibited the most dynamic concentration changes in response to altered conditions in this study. Fifteen of the 16 endometabolites contain the heteroatoms nitrogen, phosphorus, and/or sulfur and may also be valuable as organic nutrient sources to bacteria.

We found isoleucine and leucine to increase in concentration with *T. pseudonana* acclimation temperature (Fig. 2b) yet were among the more slowly assimilated metabolites by *R. pomeroyi* (Fig. 5). Thus dynamic shifts in substrate concentration overlain on varying abilities of heterotrophic bacteria to compete for substrates (11, 12, 55), adds a new level of complexity to our understanding of the ocean’s microbe-metabolite network. Substantial changes in the chemical makeup of labile marine DOC over both long and short time frames have implications for rates and efficiencies of bacterial carbon processing in the surface ocean.

## MATERIALS AND METHODS

### Culture conditions

Axenic cultures of the diatom *Thalassiosira pseudonana* CCMP1355 (National Center for Marine Algae) were acclimated to three temperature conditions (below optimal: 14°C, optimal: 20°C, and above optimal: 28°C) for 3 months with weekly transfers (~120 generations). These temperatures were selected based on previous growth rate measurements of the diatom strain across a range of temperatures (22). Diatoms were cultured in L1 medium under nutrient-replete conditions (883 µM NO_3_, 36 µM PO_4_) (56) at a salinity of 35 (57) in acid-washed glass containers under 120 µmol photons m^−2^ s^−1^ (ULM-500 Light Meter, Walz) with a 16:8 h light:dark cycle. The starter cultures were checked for purity at the initiation of the acclimation and at each weekly transfer by plating aliquots on ½ YTSS medium (58).

For the main experiment, 39 culture flasks (1.9 L Nunc polystyrene flasks) were prepared with 1 L of modified L1 medium with NaH^13^CO_3_. *T. pseudonana* was inoculated into 36 flasks, 12 per acclimation temperature, at ~3 × 10^4^ cells mL^−1^. For the 12 co-culture treatment flasks, B_12_ was limited through three weekly transfers into L1 medium with 100-fold decreased B_12_ concentration. These cultures were inoculated with alphaproteobacterium *Ruegeria pomeroyi* DSS-3, representing a bacterial taxon typically associated with phytoplankton blooms (12). Prior to inoculation, the bacterium was grown overnight in ½ YTSS medium, harvested in exponential growth phase, and washed five times in sterile artificial seawater at 6,000 relative centrifuge force (RCF). Bacteria were inoculated into the 12 co-culture flasks at ~1 × 10^5^ cells mL^−1^, with four replicates per acclimation temperature. Co-culture medium had no added vitamin B_12_ (which was instead provided by *R. pomeroyi*); axenic culture medium had B_12_ added to a final concentration of 0.37 nM (Fig. 1). A flask containing medium but no microbes was established for each treatment as a control for metabolite analysis. *T. pseudonana* and *R. pomeroyi* cultures were checked for purity at the initiation and termination of the experiment by plating on ½ YTSS and also by inspection of flow cytometry data to confirm the absence of bacterial signatures (Fig. S3).

Flasks were harvested at late exponential growth phase on days 3 (28°C), 4 (20°C), and 6 (14°C), with sampling occurring 7 h into the light cycle (Fig. 1, *n* = 2). The axenic flasks (*n* = 24, 600 mL) were immediately filtered onto 2.0 µm-pore-size Isopore filters (Millipore, Burlington, MA) to collect diatom cells, and filters were stored at −80°C for analysis of axenic endometabolomes (12 filters) and as metabolite sources in the drawdown experiment (12 filters). The co-culture flasks (*n* = 12) were subsampled by filtration onto 2.0 µm-pore-size Isopore filters for diatom RNA extraction (300 mL), diatom endometabolite analysis (600 mL), and pigment analysis (50 mL). Medium-only controls (without microbes) were similarly filtered and stored as background samples for endometabolite analysis. RNA filters were immediately flash-frozen in liquid nitrogen and together with endometabolite analysis filters stored at −80°C until processing; pigment analysis filters were stored at −20°C.

### Growth, pigments, and photosynthetic parameters

Diatom growth was measured using a Beckman Z2 Coulter Counter, with 1 mL samples collected daily from two replicates of each temperature. All samples were diluted 10× or 100× prior to analysis on the Coulter Counter to obtain a diatom cell density in a measurable range (10^3^–10^5^ cells mL^−1^). For cell enumeration at inoculation and harvest, cells were fixed with glutaraldehyde (1% final concentration) and stored overnight at 4°C and then at −80°C. Cells were stained with SYBR Green (Life Technologies) and analyzed by flow cytometry (NovoCyte Quanteon, Agilent Technologies, Inc., USA). Diatom growth rates were calculated from time of inoculation until harvest as *μ* = ln(*N*_2_/*N*_1_)/(*t*_2_ − *t*_1_), where *N* is cells mL^−1^ and *t* is time in days. Diatom cell size at harvest was determined by Coulter Counter.

Diatom cells were analyzed for pigment composition by extracting in 2 mL 90% acetone at 4°C for 24 h. Tubes were then centrifuged at 900 RFC for 1.3 min (Thermo Sorvall Legend X1R), and the supernatant was analyzed with a UV Vis spectrophotometer. The composition and concentration of these pigments were determined from an absorbance spectrum of 350–750 nm (59). Photosynthetic capacity (Fv/Fm) was obtained from 5 mL subsamples by analysis on a Satlantic FIRe Fluorometric System (blue light) within an hour of collection.

### Diatom endometabolite analysis

Filters for diatom endometabolite analysis were transferred into 15 mL of ultrapure MilliQ water in 50 mL tubes and sonicated in an ice bath for 7 min (50 s on, 10 s off, duty cycles 60, ~60 µm amplitude; Branson SLPe model) to resuspend the cells. The liquid fraction was collected in new tubes and the procedure repeated three times, after which fractions were combined and stored at −80°C until further processing. At analysis, samples were lyophilized (Labconco, Kansas City, MO, USA) and pellets mixed with 600 µL of phosphate buffer (30 mM phosphate in deuterated water, pH 7.4) and 1 mM of internal standard 2,2-dimethyl-2-silapentane-5-sulfonate. Samples were vortexed for 5 min and centrifuged at 20,800 RCF for 10 min, and supernatants were transferred to 5 mm NMR tubes (Bruker, Billerica, MA, USA). Extraction and buffer blank controls were also prepared. A pooled control sample used for annotation was prepared by combining aliquots of all samples and sample processing was carried out at 4°C.

Metabolites were analyzed by NMR spectroscopy using a 600 MHz AVANCE III HD instrument (Bruker) equipped with a 5 mm TCI cryoprobe and pulse programs of ^1^H-^13^C heteronuclear single quantum correlation (HSQC, hsqcetgpprsisp2.2 by Bruker nomenclature) and ^1^H-^13^C HSQC-total correlation spectroscopy (HSQC-TOCSY, hsqcdietgpsisp.2). TopSpin (Bruker) version 3.5 was used for NMR operation. Data were processed by NMRPipe (60). Peak intensity was extracted by rNMR version 1.11 (61), and data were analyzed by MATLAB (MathWorks) version R2023b. Peak intensity was normalized by biovolume and auto-scaled. Metabolites were annotated based on chemical shift (HSQC) and correlation information (HSQC-TOCSY). Chemical shift values for candidate peaks were obtained from the Biological Magnetic Resonance Data Bank (BRMB) (62) and the Human Metabolome Database (63), and raw reference spectra from BMRB were used for validation. Four compounds of interest that are not in these databases were annotated using literature values (homarine based on Boroujerdi et al. [64]; 2,3-dihydroxypropane-1-sulfonate [DHPS], dimethylsulfoniopropionate [DMSP], and β-1,3-glucan based on Uchimiya et al. [65]). A confidence level of annotation was assigned to each metabolite, where 1 = putative identification with functional group information; 2 = partially matched to HSQC chemical shift information in the databases or literature; 3 = fully matched to HSQC chemical shift; 4 = fully matched to HSQC chemical shift and validated by HSQC-TOCSY; 5 = validated by a spiking experiment (Table S8). All raw spectra, NMRPipe scripts for spectrum processing, and MATLAB scripts for data analysis are deposited in Metabolomics Workbench (66) under Project ID PR001837.

### Diatom mRNA analysis

RNA was extracted from filters using the ZymoBIOMICS RNA Miniprep Kit (Zymo Research, Irvine, CA, USA) according to the manufacturer’s protocol. Stranded RNAseq libraries were prepared by the Joint Genome Institute (JGI) and sequenced on a NovaSeq (Illumina). Reads were filtered and trimmed using the JGI QC pipeline, followed by evaluation of artifact sequences by kmer matching (kmer = 25) using BBDuk, allowing one mismatch; detected artifacts were trimmed from the 3′end of the reads. Quality trimming was performed using the phred trimming method set at Q6 and reads under the minimum length of 25 bases or 1/3 of the original read length were removed. Filtered reads from each library were aligned to the reference genome using HISAT2 version 2.2.0 and strand-specific coverage bigwig files were generated using deepTools v3.1, and FeatureCounts was used to generate gene counts. *T. pseudonana* gene annotations and biosynthesis pathways were based on BioCyc (67) and PhyloDB (68).

### Metabolite lability experiment

Diatom cells harvested from the 12 axenic flasks, 4 at each temperature (Fig. 1), were rinsed from thawed filters into 10 mL sterile L1 medium, probe sonicated on ice for 20 min to lyse cells (duty cycles 60, ~ 60 µm amplitude; Branson SLPe model), and passed through pre-combusted GF/F filters. A 0.5 mL subsample of each filtered, concentrated diatom endometabolome was diluted 100-fold with fresh L1 medium and acidified with 25 µl 12 M HCl. Samples were analyzed for DOC concentration on a Shimadzu TOC-L total organic carbon analyzer coupled to a TNM-L analyzer (Mass Spectrometry Lab, Woods Hole Oceanographic Institution). Milli-Q water blanks and standard curves of potassium hydrogen phthalate and potassium nitrate were run and comparisons were made daily to standards (obtained from D. Hansell, University of Miami). To assess the lability of the compounds analyzed by NMR, a 2.7 mL aliquot of each concentrated diatom endometabolome and a blank with no endometabolome addition were inoculated with *R. pomeroyi* (~3 × 10^7^ cells mL^−1^) and grown for 10 h with shaking at 30°C in dark conditions. Subsamples of 1 mL were collected at inoculation and again at 10 h for metabolite quantification. From these, 540 µL were mixed with 60 µL phosphate buffer and transferred to NMR tubes, and spectra were collected using the HSQC procedure described above. One medium blank was also included. Experimental and spectrum processing settings were those described above, except TopSpin version 3.6.4 was used for NMR operation and NMRPipe was run on NMRbox (69). Endometabolite peak intensity was normalized to diatom biovolume at time of harvest. The mean lifetime (*t*) of each metabolite was calculated as *t* = 1 /l, where *l* is the specific removal rate from bacterial uptake (units of per hour). For metabolites with approximate zero concentration at the 10 h sample time (set at ≤2% of initial), these calculations represent the maximum mean lifetime.

### Data analysis

Statistical significance of the response of diatom endometabolome composition under different acclimation temperatures was determined by the Kruskal Wallis Test with Mann Whitney *U* Tests for post hoc pairwise comparisons. Statistical significance of endometabolite concentrations in axenic versus co-culture endometabolomes was determined by Student’s *t*-tests with Benjamini-Hochberg correction for multiple hypothesis testing. Analysis for gene expression differences between *T. pseudonana* transcriptomes was carried out using DESeq2 (version 1.28.1). Sample numbers (*n*) were 3 or 4 for all analyses, and *P* value cutoffs were ≤0.05. Genes with significant relative differences between acclimation temperatures 14°C and 28°C are given in Table S6. Genes with significant differences between co-culture and axenic treatments for 14°C, 20°C, and 28°C are given in Tables S3 to S5.

## Data Availability

Raw and annotated *T. pseudonana* transcriptome data are available through the JGI Genome Portal, proposal ID 506891 (https://genome.jgi.doe.gov/portal/) and BCO-DMO (DOI: 10.26008/1912/bco-dmo.905306.1). Metabolomics data and associated sample preparation protocols and NMR analysis and processing parameters are deposited at the Metabolomics Workbench Data Repository under Project ID PR001837 (DOI: 10.21228/M88B0T) and BCO-DMO (DOI: 10.26008/1912/bco-dmo.928203.1).

## SUPPLEMENTAL MATERIAL

Supplemental Figures msystems.01036-25-s0001.pdf Fig. S1 to S3.

Supplemental Tables msystems.01036-25-s0002.xlsx Tables S1 to S8.

ASM does not own the copyrights to Supplemental Material that may be linked to, or accessed through, an article. The authors have granted ASM a non-exclusive, world-wide license to publish the Supplemental Material files. Please contact the corresponding author directly for reuse.
