# Supplementary material for: Dynamic reworking of marine diatom endometabolomes in response to temperature and a model bacterium
Source: mSystems. 2025 Dec 15;11(1):e01036-25. doi: 10.1128/msystems.01036-25 (PMC12817926; doi:10.1128/msystems.01036-25)
Supplement: Supplemental Figures — Fig. S1 to S3. [file msystems.01036-25-s0001.pdf]

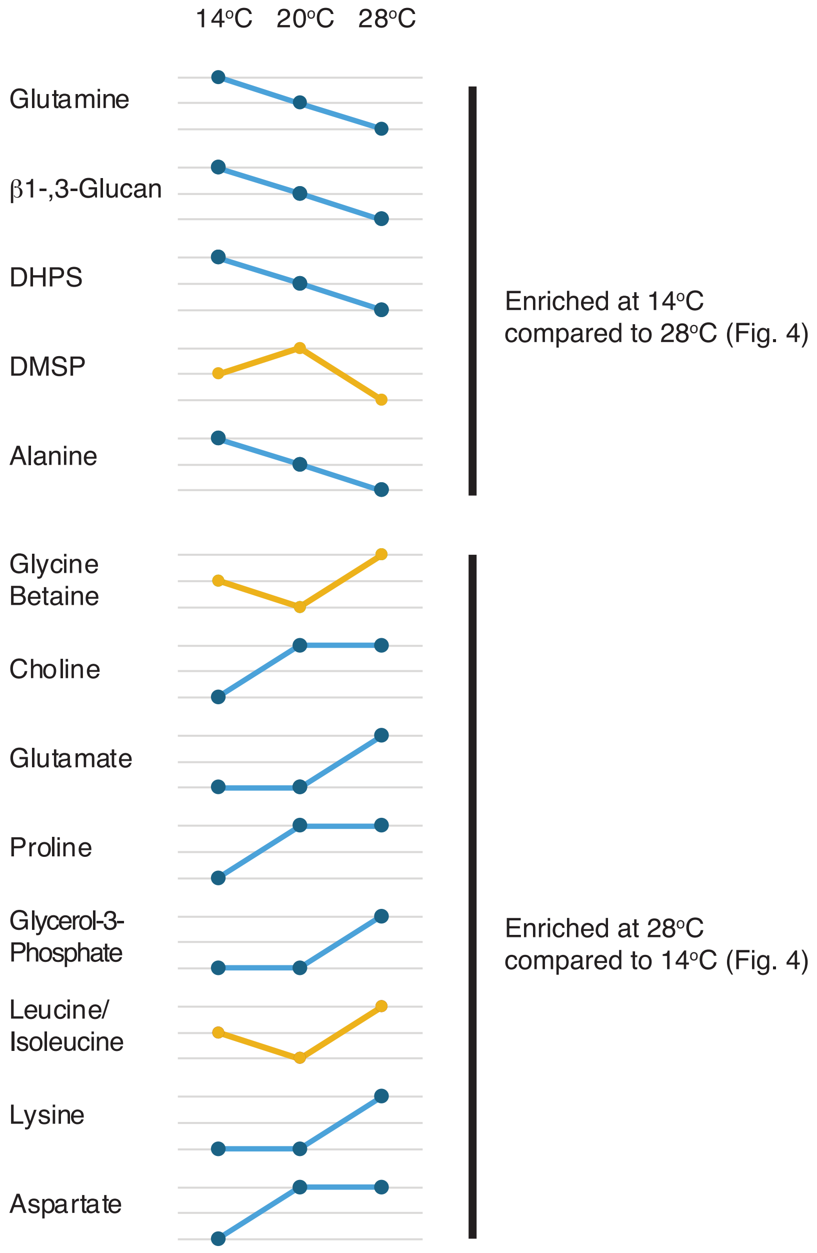

Figure S1. Relative transcript abundance mapping to thirteen tentatively identified metabolite biosynthesis pathways in temperature acclimated *T. pseudonana* strains in co-culture with *R. pomeroyi*. Each line indicates the ranked outcome of gene expression at each temperature (gray lines indicate lowest, middle, highest expression) from the three pairwise comparisons of biosynthetic pathway expression. Ranking is based on the majority of significantly enriched genes in a pathway, as in Fig. 4. The 20°C comparisons (14°C versus 20°C; 20°C versus 28°C) that are inconsistent with the 14°C versus 28°C comparison shown in Fig. 4 are indicated with yellow lines (3 out of 13).

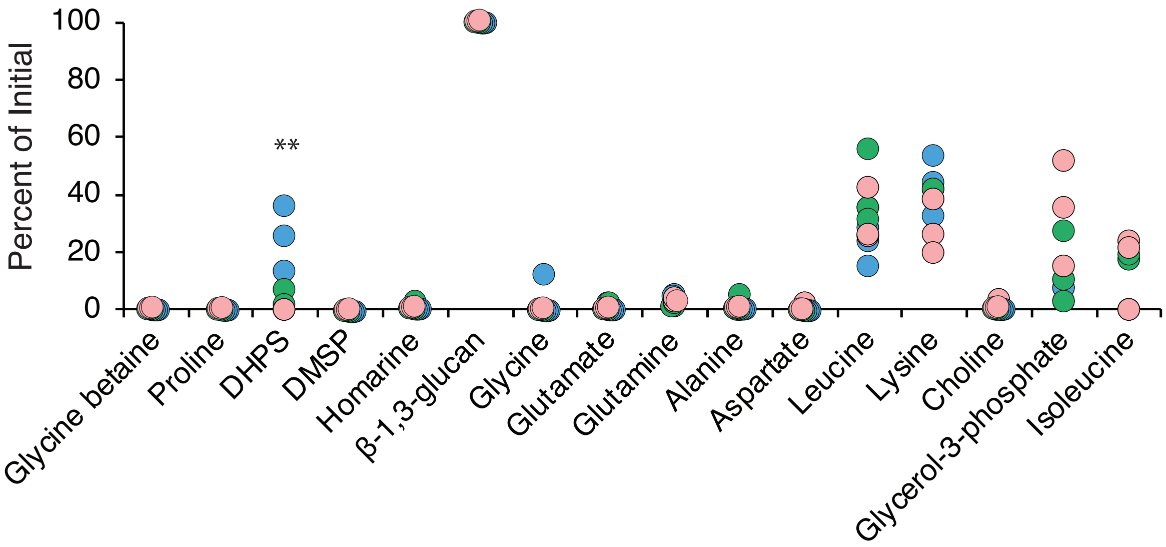

Figure S2. Drawdown of 16 *T. pseudonana* endometablites by *R. pomeroyi* over a 10 h incubation at 30°C. Endmetabolites were extracted from diatom strains acclimated at 14°C (blue), 20°C (green), and 28°C (pink) (Kruskal Wallis test, $p \leq 0.01$). Asterisks indicate a significantly lower drawdown rate of DHPS in cell lysates at 14°C relative to 20°C and 28°C. β-1,3-glucan was not taken up by the bacterium. DHPS, 2,3-dihydroxypropane-1-sulfonate. DMSP, dimethylsulfoniopropionate.

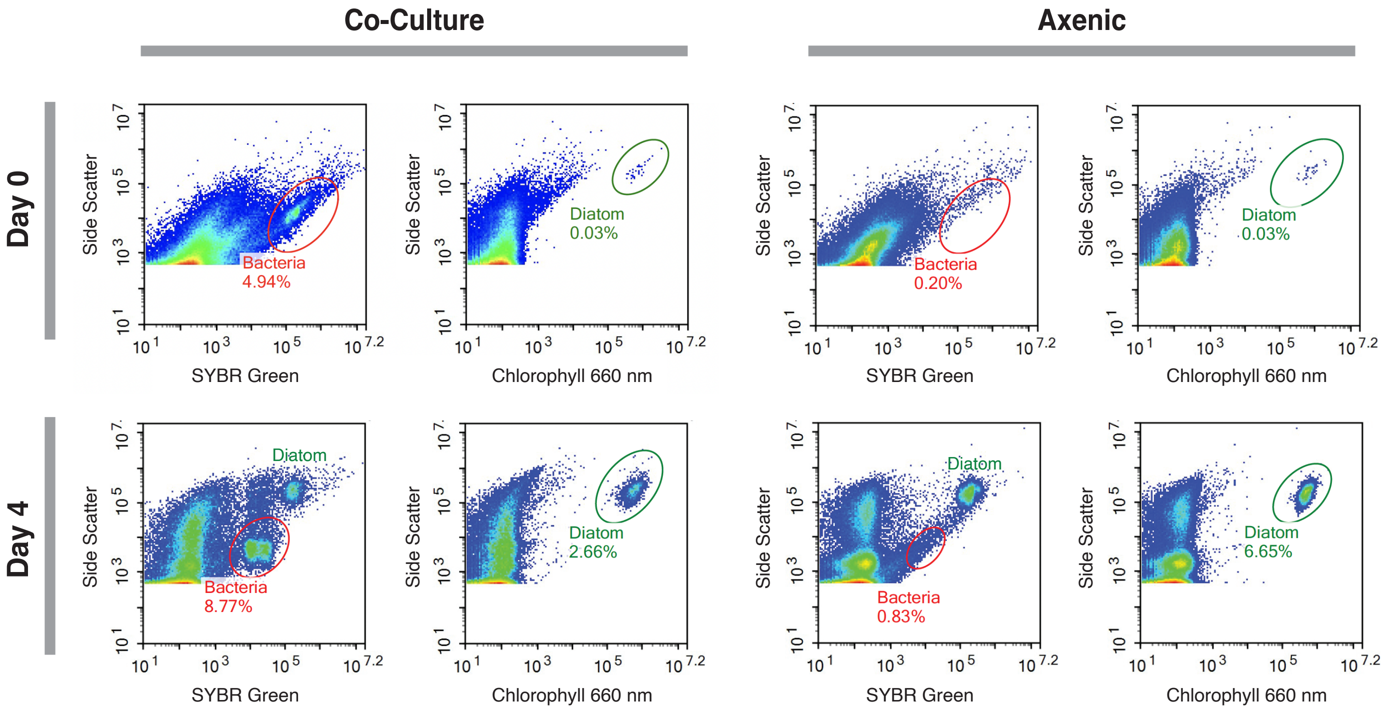

Figure S3. Flow cytometry data confirms axenicity. Each pair of flow cytometry plots shows the bacterial population (red circle; SYBR Green fluorescence) and diatom population (green circle; chlorophyll autofluorescence), and gives the precent of particles within each gate compared to total particles detected. The low-fluorescing background particles represent cellular fragments and non-cellular debris. Co-culture plots show bacterial inoculum at Day 0 and final population at exponential phase harvest (Day 4). The axenic plots show the absence of a bacterial population at both time points. Plating culture aliquots on 1/2 YTSS agar at initial and final time points similarly indicated that axenic cultures were not contaminated. This example is from a 20°C acclimation temperature replicate.
